# Cysteamine HCl Administration Impedes Motor and Olfactory Functions, Accompanied by a Reduced Number of Dopaminergic Neurons, in Experimental Mice: A Preclinical Mimetic Relevant to Parkinson’s Disease

**DOI:** 10.3390/brainsci14070632

**Published:** 2024-06-24

**Authors:** Divya Bharathi Selvaraj, Anusiya Panneerselvam, Jemi Feiona Vergil Andrews, Mahesh Kandasamy

**Affiliations:** 1 Laboratory of Stem Cells and Neuroregeneration, Department of Animal Science, School of Life Sciences, Bharathidasan University, Tiruchirappalli 620024, India; divyabharathimsr26@gmail.com (D.B.S.); jemiandrews1996@gmail.com (J.F.V.A.); 2 Department of Biotechnology, Bharathidasan University, Tiruchirappalli 620024, India; anusiyabt11@gmail.com; 3 University Grants Commission-Faculty Recharge Programme (UGC-FRP), New Delhi 110002, India

**Keywords:** cysteamine HCl, motor coordination, olfaction, rotarod, tyrosine hydroxylase, Parkinsonism

## Abstract

Cysteamine hydrochloride (Cys-HCl) has been established as a potent ulcerogenic agent of the gastrointestinal (GI) system. GI dysfunction and olfactory deficits are the most common clinical symptoms of many movement disorders, including Parkinson’s disease (PD). Cys-HCl has been shown to interfere with dopamine, a neurotransmitter crucial for motor, olfactory, and cognitive functions. However, the reports on the effect of Cys-HCl treatment on the behavioral aspects and functions of the dopamine system appear to be inconsistent. Therefore, we revisited the impact of Cys-HCl on the motor function in experimental mice using a battery of behavioral tests, such as the pole test (PT), beam-walking test (BWT), and rotarod test (RDT), while the olfactory ability and cognitive functions were examined through the buried-food test (BFT) and Y-maze test. Furthermore, we investigated the effect of Cys-HCl on the number of dopaminergic tyrosine hydroxylase (TH)-positive cells in the substantia nigra (SN) and olfactory bulb (OB) of the experimental mice using immunohistochemistry. The results revealed that Cys-HCl administration in the mice induced significant impairments in their motor balance and coordination, as their movement-related performances were markedly reduced in terms of the behavioral tasks. Mice exposed to Cys-HCl showed pronounced reductions in their odor discrimination abilities as well as cognitive impairments. Strikingly, the number of TH-positive neurons was found to be reduced in the SN and OB of the Cys-HCl-treated group, which is a bonafide neuropathogenic hallmark of PD. This study highlights the potential neurotoxic effects of Cys-HCl in experimental brains and suggests further investigation into its role in the pathogenesis of Parkinsonism.

## 1. Introduction

Cysteamine hydrochloride (Cys-HCl) is a potent ulcerogenic agent, as it stimulates the hypersecretion of hydrochloric acid (HCl) in the stomach, leading to gastritis and peptic ulcers [1]. The physiological levels of intestinal alkaline phosphatase play a crucial role in maintaining the homeostasis and supporting the immune defense mechanism of the gastrointestinal (GI) tract [2]. Studies have shown that Cys-HCl can counteract the activity of alkaline phosphatase in mucosal cells of the gut. When alkaline phosphatase activity is inhibited in the GI tract, it can lead to dysbiosis and prolonged intestinal inflammation, followed by the systemic activation of immune cells [3]. The pro-inflammatory factors discharged from the activated immune cells in the circulation can subsequently produce a negative impact on the brain [4]. As the aberrant circadian rhythm often coincides with GI disorders, the Cys-HCl-induced pathophysiology of the GI tract can result in sleep disorders leading to mood, neurocognitive, and neurological disorders [5,6]. Moreover, ulceration in the GI tract caused by Cys-HCl can also deteriorate the gut–brain axis, contributing to behavioral and neurophysiological abnormalities [7,8]. As Cys-HCl can cross the blood–brain barrier (BBB), it has become more apparent that it can also cause anxiety and neurocognitive impairments [9]. Furthermore, few animal studies have shown that Cys-HCl treatment can induce neurotoxicological effects similar to those provoked by 1-methyl-4-phenyl-1,2,3,6-tetrahydropyridine (MPTP), an authenticated neurotoxin known to induce the clinical symptoms of Parkinson’s disease (PD) [7,10]. Cys-HCl competitively antagonizes various ion channels and neurotransmitter systems due to its structural resemblance to ethylamine, a precursor of norepinephrine, dopamine, and serotonin, which serve as crucial neuromodulators of the stress response, motor function, mood, and cognitive functions [10,11]. In particular, Cys-HCl-mediated GI dysfunction in association with the alteration of the dopamine system has been an intense research focus [7]. Meanwhile, the deleterious effects of Cys-HCl on neuroplasticity responsible for movement disorders have also been recognized, but to a lesser extent [12]. Considering its dopamine depletion capacity, few studies have indicated that Cys-HCl treatment induces cognitive deficits, while its effects on locomotive behavior remain inconclusive [9,12,13]. Notably, reduced levels of dopamine have been linked to the underlying causes of the movement disorders seen in prevalent neurodegenerative disorders, such as PD [14,15]. Notably, many movement disorders have also been characterized by olfactory deficits, suggesting a potential neuropathogenic overlap between movement disorders and OB deficits [15,16,17]. Hence, it can be proposed that Cys-HCl treatment may be associated with the onset and progression of movement disorders and olfactory deficiencies due to its negative impact on the brain’s dopamine system. Despite accumulating evidence suggesting a potential link between Cys-HCl treatment and neurological functions, reports on its effects on the motor function and dopaminergic system remain inconsistent. Moreover, the impact of Cys-HCl on olfactory functions has not been elucidated. Therefore, we revisited the effects of Cys-HCl administration on the motor, cognitive, and olfactory functions in experimental mice using a battery of behavioral paradigms. We also extended our investigations to assess the impact of Cys-HCl on the density of tyrosine hydroxylase (TH)-positive cells, the main dopamine-producing cells in the substantia nigra (SN), and the glomerular layer of the olfactory bulb (OB), using immunohistochemistry.

## 2. Materials and Methods

### 2.1. Animals and Treatment

Four–five-month-old male BALB/c mice were procured from the Biogen laboratory, Bangalore, India. The animals were maintained under identical conditions of a temperature of 22–24 °C and a 12 hour (h) light–dark cycle in the animal house facility at Bharathidasan University, with free access to food and water, and their body weights were monitored regularly. The mice (N = 12) were divided into two experimental groups: the control group (N = 6), which received intraperitoneal injections of sterile water, and the Cys-HCl group (N = 6), which received intraperitoneal injections of 60 milligrams (mg)/kilogram (kg) of body weight (BW) of Cys-HCl for three alternative days for a total of three doses. The dose and concentration of Cys-HCl, as well as the choice of vehicle, were as described earlier [18]. After 14 days, the animals were exposed to the respective behavioral apparatuses and arenas for acclimatization and basic training prior to each experiment. Accordingly, all the mice were subjected to behavioral tests, such as the pole test, beam-walking test, rotarod test, buried-food test, and Y-maze test, as per previous studies, with some modifications, and they were then perfused. Each animal in both groups underwent a minimum of 3 trials in all the behavior experiments, and the average values were taken for the comparison. After 30 days from the last injection of Cys-HCl, the animals were sacrificed, and the brains were collected and further processed for immunohistochemical analysis. All animal experiments were conducted following approval from the Institutional Animal Ethical Committee (IAEC), Bharathidasan University, under the regulations of the Committee for the Purpose of Control and Supervision of Experiments on Animal (CPCSEA), India (Ref No. BDU/IAEC/P10/2019, dated 30 November 2019).

### 2.2. Pole Test

A vertical pole measuring 60 centimeter (cm) in height and 1.9 cm diameter was used to assess the motor function and coordination in the experimental mice. The base of the pole was positioned in a new cage, which was filled with fresh husk bedding. Each animal was gently placed onto the top of the pole, facing upward. The time taken to reorient themselves downwards (T-time) and the total time taken to descend to the bottom of the pole (T-total) were measured. Initially, during the pole test, the animals either did not move, took a long time, or were unable to complete the task in the first two trials. Subsequently, the animals in the control group completed the task promptly, while the animals in the treatment group completed the task with some delay compared to the control group. All animals underwent six trials in total. For calculation purposes, the latency to complete the task was measured using the data from the six animals in each group that successfully completed 3 trials within a 3 min duration [19].

### 2.3. Beam-Walking Test

A beam with a 1-m length and 1.9 cm width raised 50 cm above the ground level was used to study the motor coordination and gait in the experimental mice. An empty cage was placed at the endpoint. The ground under the beam was spread with a spongy material to avoid accidental injuries. Initially, the mice were trained to acclimatize with an elevated beam in the training session. Then, each animal was gently released on the beam, and the time taken to cross the beam and the number of slips were noted down. A total of three successful trials from each animal were considered to calculate the latency to complete the task [20].

### 2.4. Rotarod Test

Next, the motor coordination and balance in the experimental mice were evaluated using a semiautomated rotarod apparatus (Inco, Ambala, India). Initially, each mouse was placed onto the rotating rod and trained to walk at a speed of 3 revolutions per minute (rpm). During the test phase, the speed was gradually raised from 3 rpm to 25 rpm for every 10 s within the span of 2 min, and the latency to falling from the rotating rod was recorded [21].

### 2.5. Buried-Food Test

In order to measure the olfaction ability, the experimental mice were subjected to the buried-food test. Prior to the experiments, the food pellets were removed from their home cage, and the animals were deprived of food for 48 h. Then, the animals were exposed to a tiny portion of chocolate-flavored food. Two hours later, the chocolate-flavored food was kept underneath at the mid-end of a rectangular box filled with clean bedding 3 inches in height. The test arena was digitally divided into two zones using the SMART 3.0 video-tracking module. While the blue-colored zone 1 designated the entire area, the food-buried area was marked as green-colored zone 2. The animals were released into the arena and given 5 min to explore for three consecutive trials. The time taken by the animals to collect the buried food was recorded as the latency [22].

### 2.6. Y-Maze Test

To assess the exploration-based spatial memory in the mice, a Y-maze apparatus was digitally divided into four zones, namely, zone 1, the middle area marked in the triangular-shaped pink color, the familiar areas, zone 2 and zone 3, marked in the blue color and brown color, respectively, and the unfamiliar area, zone 4, marked with the green color. In the training phase, zone 4 (green color) was closed to prevent entry. Each animal was placed in zone 1 and allowed to explore all areas for 5 min, excluding zone 4, and each received three trials. The next day, the barrier to enter zone 4 was removed, and each mouse was released into zone 1. The exploratory tendency of the animals in all the sessions was captured using the SMART 3.0 video-tracking module, and the time spent by each mouse in the unfamiliar zone 4 and the distance traveled were calculated [23].

### 2.7. Immunohistochemical Assessment of TH-Positive Dopaminergic Neurons in SN and OB

The animals were anesthetized and perfused using 0.9% saline followed by 4% paraformaldehyde (PFA) (Himedia, Mumbai, India), and the brains were post-fixed in the PFA for 24 h. Further, the brains were stored in a 30% sucrose (SRL, Mumbai, India) solution at 4 °C for a week. Then, the brains were cut into 30-micrometer (µm) sagittal sections using a dry-ice-based sliding microtome (Weswox, Ambala, India) and were stored in a cryoprotectant solution. For the immunohistochemical analysis, 1 out of 12 brain sections was placed in a 12-well plate (Tarson, Kolkata, India) and washed thrice with 1× Tris-buffered saline (TBS) for 10 min. For the antigen retrieval, the sections were treated with 10 millimolars (mM) of sodium citrate buffer (Thermo Fisher Scientific, Waltham, MA, USA) at 65 °C for 90 min. After incubation, the sections were washed thrice with 1× TBS for 10 min. Then, the sections were blocked with 3% bovine serum albumin (BSA) (Himedia, Mumbai, India) for an hour before being treated with the primary antibody. The brain sections were transferred to a solution containing a rabbit α-TH antibody with a dilution of 1:250 (Cell Signaling Technology, Danvers, MA, USA) and incubated at 4 °C for 48 h. After incubation with the primary antibody, the sections were washed thrice with 1× TBS for 10 min each. Further, the brain sections were incubated with a goat α rabbit DyLight^TM^ 594 secondary antibody with a dilution of 1:500 (Novus Biologicals, Denver, CO, USA) and kept at 4 °C for 24 h. The next day, the sections were washed twice with 1× TBS for 10 min. Further, the sections were placed on the microscopic slides and dried. The next day, the specimens were sealed with ProLong^TM^ Glass antifade mountant (Thermo Fisher Scientific, Waltham, MA, USA) and dried overnight. The slides were blind-coded, and the brain sections were analyzed and photographed using a fluorescence microscope (DM750, Leica Microsystems, Wetzler, Germany). The numbers of TH-positive cells were estimated in the SN and glomerular layer of the OB in five brain sections from each animal using the ImageJ (Version 1.54) plugin with a cell counter. The average number of cells per section was calculated and compared among the control and Cys-HCl-treated groups.

### 2.8. Statistical Analysis

The values are represented as means ± standard deviations (SDs) and are presented along with the *p* and t values. All the statistical analyses were performed using the Students’ *t*-test in GraphPad Prism. The statistical significance was assumed at *p* < 0.05 unless otherwise indicated. The asterisk symbols on top of the bar graphs indicate statistical significance, with * denoting *p* < 0.05, ** denoting *p* < 0.01, and *** denoting *p* < 0.001.

## 3. Results

### 3.1. Cys-HCl-Treated Mice Exhibited Delayed Motor Functions during Pole Test

**Figure 1:**
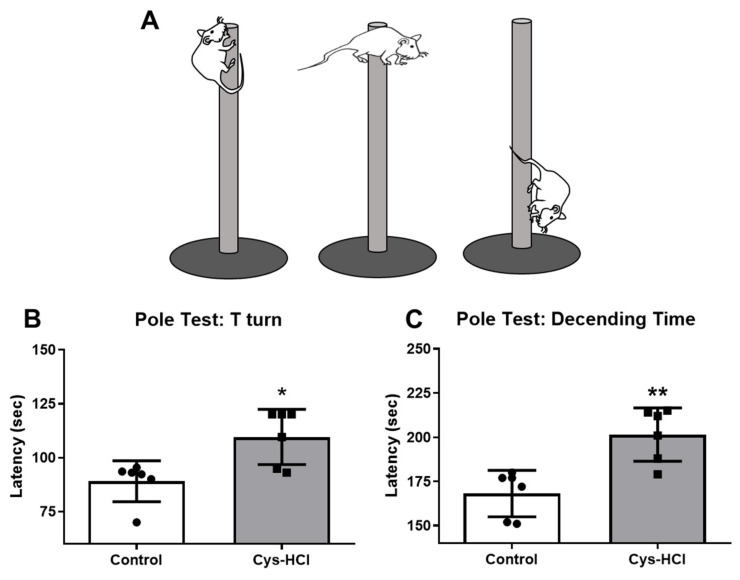
Impaired motor coordination upon Cys-HCl treatment in the pole test. (**A**) Diagrammatic representation of motor activities of animals in the pole test; (**B**) latency of T-turn and (**C**) latency to reach the bottom of the pole by the experimental animals. * denotes *p* < 0.05 and ** denotes *p* < 0.01.

The pole test determined the movement disorder in the experimental animals, during which the time taken by the animals to face the downward position was considered the T-time. The Cys-HCl-treated group displayed a significant delay in their latency to orient downward when compared with the control group (control: 89.05 ± 9.50 vs. Cys-HCl: 110 ± 13; *p* value = 0.0103; t value = 3.153). Subsequently, the latency to reach the base of the pole was significantly increased in the Cys-HCl group compared with that of the control group (control: 168 ± 13 vs. Cys-HCl: 201 ± 15; *p* value = 0.0022; t value = 4.078), suggesting Cys-HCl-mediated delays in the motor performances of the experimental animals (Figure 1).

### 3.2. Cys-HCl-Treated Mice Showed Impaired Motor Coordination and Balance in Beam-Walking Test

**Figure 2:**
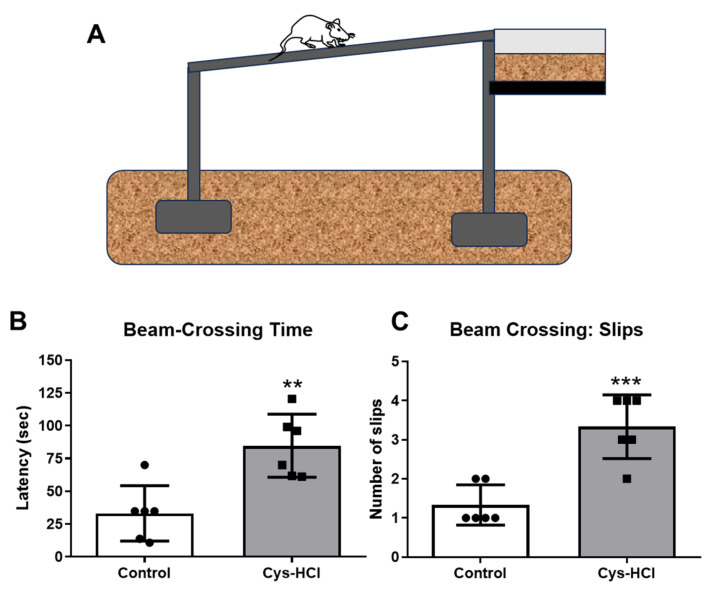
Cys-HCl treatment impaired motor coordination and gait in the beam-walking test. (**A**) Image depicting the beam-walking test; (**B**) latency to cross the beam; and (**C**) number of foot slips during the beam crossing by the experimental animals. ** denotes *p* < 0.01 and *** denotes *p* < 0.001.

The beam-walking test was employed to assess the motor coordination and balance of the experimental animals. The total time taken by the Cys-HCl-treated mice to traverse the raised beam was significantly increased when compared with that of the control group (control: 33 ± 21 vs. Cys-HCl: 88 ± 30; *p* value = 0.0005; t value = 5.071). During the beam crossing, there was a notable increase in the number of foot slips observed in the Cys-HCl-treated animals compared to the control group (control: 0.7 ± 0.8 vs. Cys-HCl: 3 ± 0.9, *p* value = 0.0028, t value = 3.940). The outcome of the balance beam test indicates that the Cys-HCl impaired both the motor coordination and balance of the experimental animals (Figure 2).

### 3.3. Cys-HCl Treatment Diminished Locomotor Behavior in Rotarod Test

**Figure 3:**
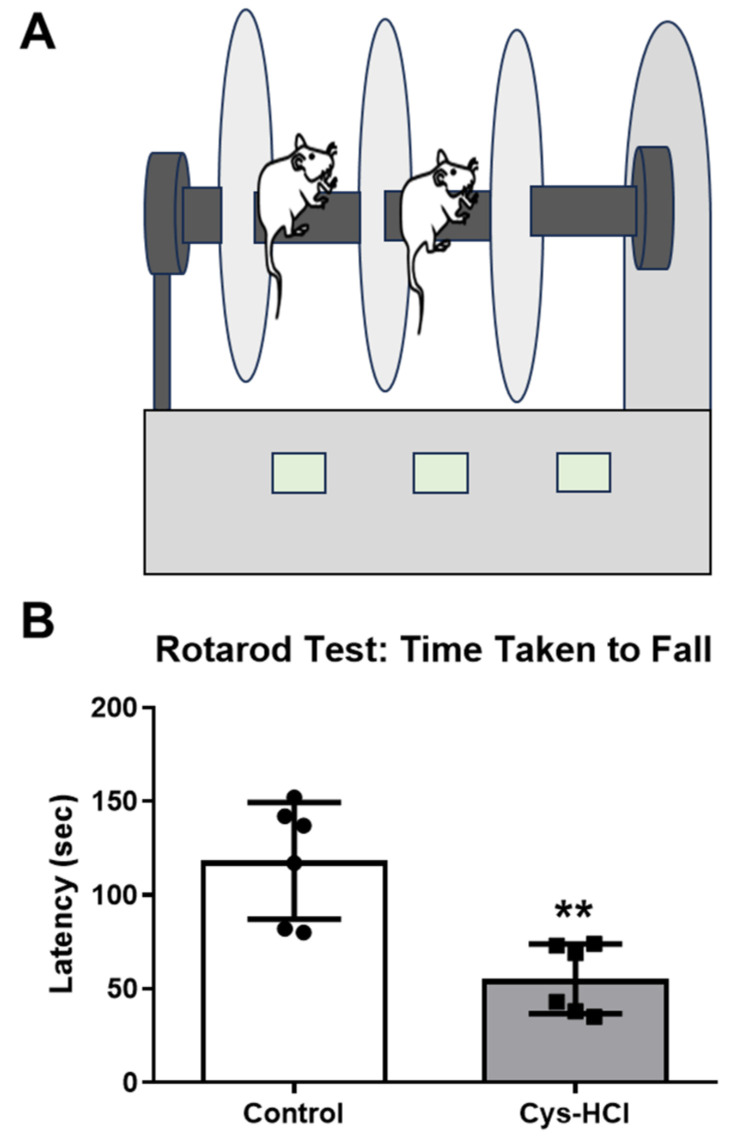
Cys-HCl administration impaired the locomotory performances of mice in the rotarod test. (**A**) Illustration of the rotarod test and (**B**) latency to falling during the rotarod test. ** denotes *p* < 0.01.

The rotarod test was used to assess the effect of the Cys-HCl on the locomotor performance, such as motor coordination- and balance-based activities. While the control group showed a better performance in the rotarod test, the latency on the rotating rod was considerably decreased in the Cys-HCl-treated animals (control: 118 ± 31 vs. Cys-HCl: 55 ± 18: *p* value = 0.0017; t value = 4.265). The results from the rotarod test specify that the Cys-HCl-treated animals were not able to stay on the rod for longer periods, demonstrating a poorer performance in terms of motor coordination, balance, and endurance. This observation lines up with the findings from the pole test and beam-walking test, which also showed significant impairments in the neurological functions responsible for movement and balance in the Cys-HCl-treated group (Figure 3).

### 3.4. Cys-HCl-Treated Animals Showed Deteriorated Olfactory Behavior

**Figure 4:**
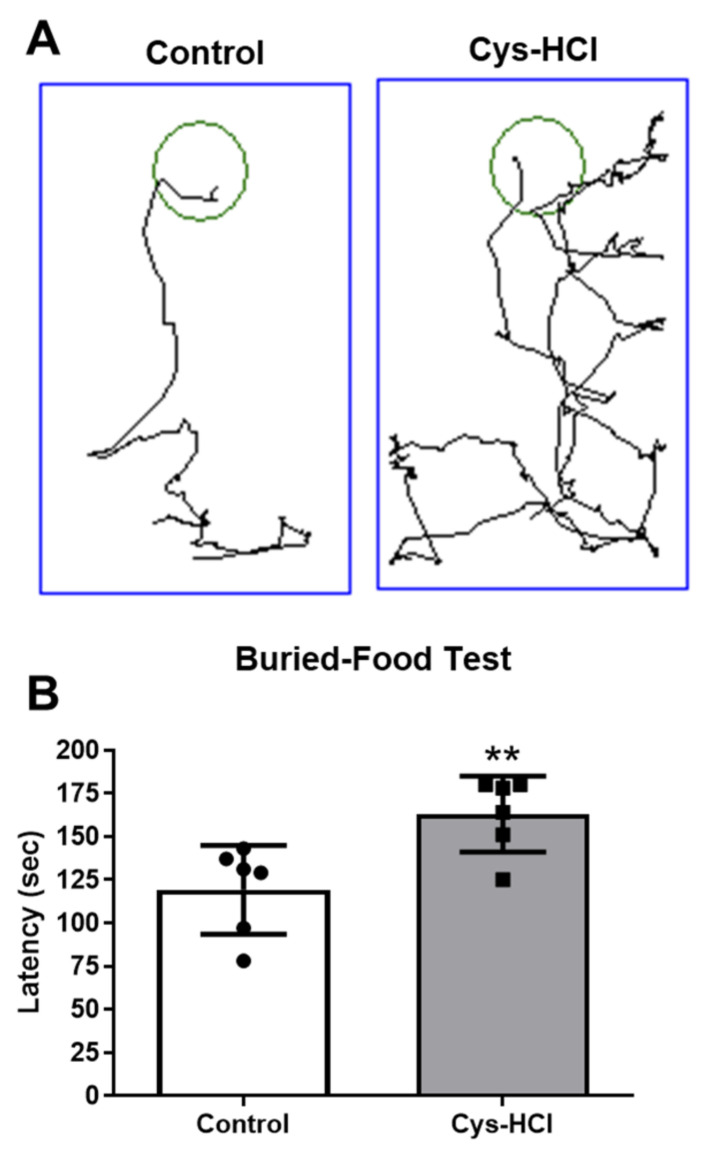
Cys-HCl treatment impeded the olfactory behavior in the buried-food test. (**A**) Representative tracking image of buried-food test of control and Cys-HCl-treated mice. The green color shows the hidden food zone, and the blue color represents the overall test arena. (**B**) Time taken to find the buried food by the experimental animals. ** denotes *p* < 0.01.

The buried-food test was conducted to assess the effect of the Cys-HCl on the olfactory function, as it indicates the ability of animals to sense buried food. The Cys-HCl-treated animals took a longer time to locate and retrieve the buried food compared to the control group (control: 116 ± 33 vs. Cys-HCl: 154 ± 42: *p* value = 0.0098; t value = 3.180). This prolonged latency indicates the impediment of Cys-HCl treatment with the neural control of olfaction, as the treated animals were less efficient at using their senses of smell to find the hidden food (Figure 4).

### 3.5. Impaired Spatial Memory in Cys-HCl Treatment during Y-Maze Test

**Figure 5:**
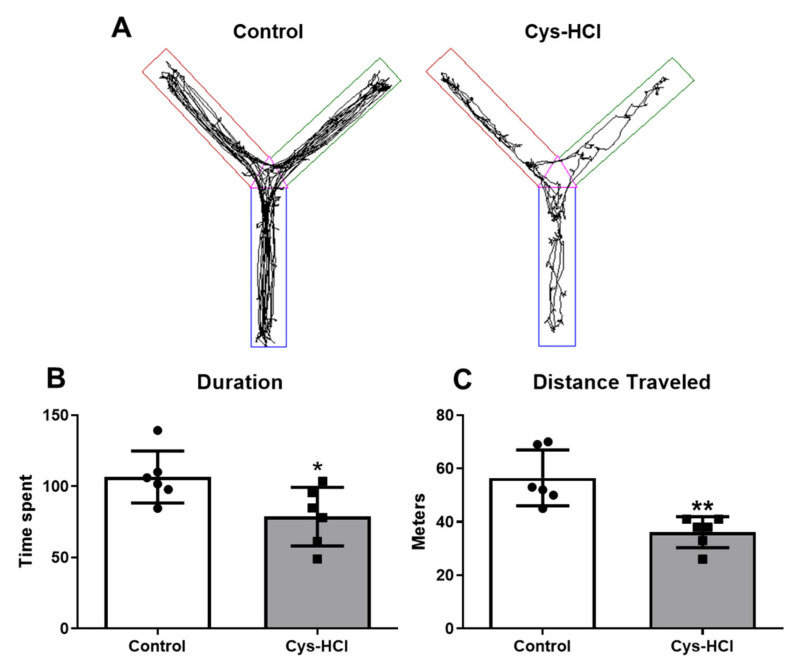
Impaired spatial memory in the Cys-HCl-treated group during the Y-maze test. (**A**) The image represents the video tracking of the control and Cys-HCl-treated mice during the Y-maze test. The pink color indicates zone 1, the green color represents zone 2, and, in the novel arm, the brown color indicates zone 3 and the blue color represents zone 4. (**B**) Time spent by the experimental animals in the novel zone. (**C**) Distance traveled by the animals during the Y-maze test. * denotes *p* < 0.05 and ** denotes < 0.01.

The Y-maze test was used to evaluate the ability of the animals to remember and explore new environments based on a spatial reference, which is indicative of their cognitive function. In this task, the control group spent a considerable amount of time exploring unfamiliar zone 4, indicating normal curiosity and memory functioning. In contrast, the Cys-HCl-treated animals spent significantly less time in unfamiliar zone 4, suggesting impaired exploration-based spatial working memory (control: 106 ± 18 vs. Cys-HCl: 79 ± 21; *p* value = 0.0326; t value = 2.479). The distance traveled by the Cys-HCl-treated mice was significantly reduced compared with that traveled by the control group (control: 56 ± 11 vs. Cys-HCl: 37 ± 5, *p* value = 0.0039; t = 3.733). This suggests that Cys-HCl could have potential adverse effects by interfering with neurocognitive functions, highlighting the need for further research into its neurotoxicological effects and the related mechanisms underlying these behavioral changes (Figure 5).

### 3.6. Cys-HCl Treatment Decreased Number of TH-Positive Cells in Substantia Nigra and Olfactory Bulb

**Figure 6:**
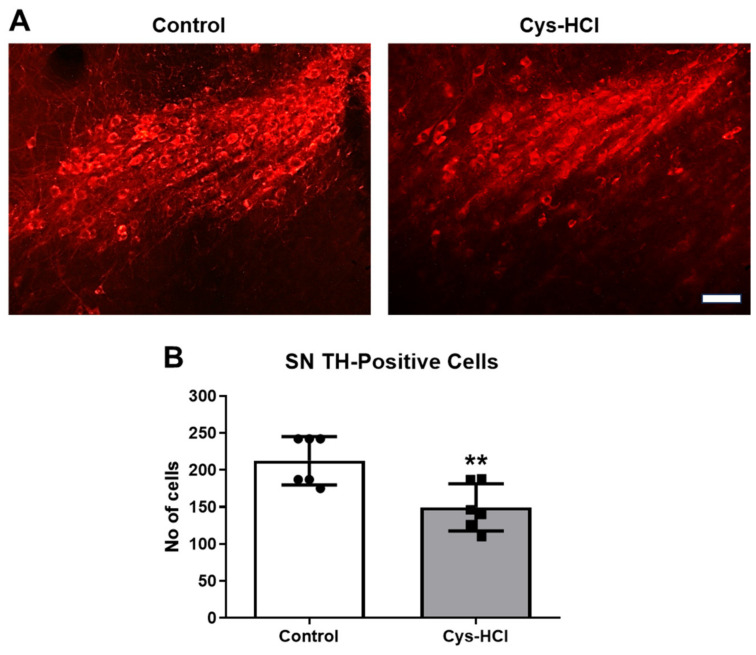
Cys-HCl treatment decreased the number of TH-positive cells in the substantia nigra. (**A**) Representative immunofluorescence images of TH-positive cells in the substantia nigra of the control and Cys-HCl-treated mice. (**B**) The number of TH-positive cells in the substantia nigra of the experimental animals. Scale bar: 25 µm. ** denotes *p* < 0.05.

**Figure 7:**
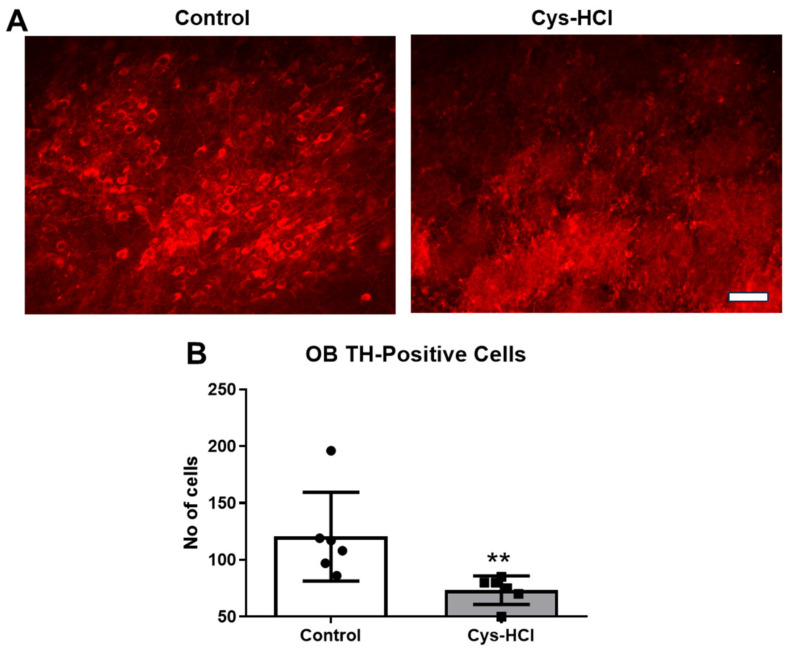
Cys-HCl treatment reduced the amount of tyrosine hydroxylase (TH) in the glomerular layer of the olfactory bulb. (**A**) Representative immunofluorescence images of TH-positive cells in the glomerular layer of the olfactory bulb of the control and Cys-HCl-treated mice. (**B**) The number of TH-positive cells in the olfactory bulb of the experimental animals. Scale bar: 25 µm. ** denotes *p* < 0.01.

TH is the rate-limiting enzyme involved in the synthesis of dopamine in the brain. TH-immunopositive cells are indicative of dopaminergic neurons in the SN and OB [24]. Dopaminergic neurons play crucial roles in various brain functions, including motor control, motivation, reward processing, and the regulation of mood, cognition, and olfaction [25]. Hence, the effect of Cys-HCl on the number of TH-positive cells was evaluated in the SN and OB. The immunohistochemical assessment revealed that the Cys-HCl treatment significantly decreased the number of TH-positive cells in both the SN (control: 212 ± 33 vs. Cys-HCl: 149 ± 32: *p* value = 0.0070; t value = 3.380) and OB (control: 121 ± 39 vs. Cys-HCl: 75 ±9: *p* value = 0.0179; t value = 2.830). This indicates that Cys-HCl treatment could adversely affect the survival of dopaminergic neurons, with potential implications for disease conditions like PD (Figure 6 and Figure 7).

## 4. Discussion

In an experimental quest to explore the pathogenic interplay between gastrointestinal (GI) disorders and neurological impairments, the current study revisited the deleterious effects of Cys-HCl, an established ulcerogenic agent, on key neurobiological aspects in experimental mice using a battery of behavioral paradigms and immunohistochemical methods. The present study demonstrates that the Cys-HCl administration induced distinguishable movement disorders, olfactory dysfunction, and signs of cognitive impairment in the experimental mice, accompanied by a marked reduction in dopamine-producing TH-positive cells in both the SN and OB. Strikingly, these findings suggest a compelling preclinical model of GI disorder induced by Cys-HCl that mirrors key symptomatic aspects of PD, including motor deficits, olfactory dysfunction, and dopaminergic neuronal loss. The use of various motor behavioral paradigms, such as the pole test and rotarod test, confirmed significant impairments in the motor coordination and reduced locomotive activity levels.

Cys-HCl is an established chemical agent used to model GI disorders in laboratory rodents [6,10]. While the exact mechanism for the ulcerogenic property of Cys-HCl is not completely understood, multiple experimental studies suggest that it hyperstimulates the secretion of HCl and gastrin in the gut, leading to inflammation in the GI tract [1]. Notably, the subcutaneous administration of Cys-HCl in experimental rodents has been reported to decrease the concentrations of somatostatin in the brain [26,27,28,29]. Somatostatin is a key hormone that interferes with the secretion of several other hormones important for growth, physiology, metabolism, and neurological functions [30]. The Cys-HCl-induced decrease in the somatostatin-positive cells in the cortical and hippocampal regions of the brains of Sprague–Dawley rats have been reported to be associated with considerable motor deficits and memory impairments [31]. Moreover, Martin-Iverson et al. demonstrated that local injections of Cys-HCl into the nucleus accumbens decreased the amphetamine-induced hyperlocomotive activities and reduced the somatostatin levels [12]. While the effects of the synergistic actions among somatostatin and dopamine on the hormonal regulation of the pituitary have been well established, classical radioimmunoassay-based experiments revealed a drastic reduction in somatostatin in the frontal cortex and hippocampus in subjects with PD [32]. Moreover, somatostatin pretreatment in experimental rat models mimicking PD has been reported to show resistance against lipopolysaccharide-induced neurodegeneration in the SN by preventing the activation of microglia and the accumulation of reactive oxygen species [33]. In a recent finding, we demonstrated that the administration of Cys-HCl induced an anxiety-like phenotype in the experimental animals in correlation with the reduced neuronal density and increased activation of microglia cells in the hippocampus [8,18]. While, in the intact brain, somatostatin plays a key role in olfaction and mood, affective disorders and olfactory dysfunctions have been established as non-motor clinical symptoms of many neurodegenerative disorders, including PD [30,34,35]. Ample experimental evidence indicates that reduced levels of somatostatin can prompt olfactory impairments and anxiety-related disorders [30,34,36]. Therefore, the Cys-HCl-induced motor, olfactory discrimination, and cognitive deficits noticed in the experimental animals could be linked, in part, to their reduced levels of somatostatin.

Numerous research studies have shown the high prevalence of peptic ulcers in patients with anxiety and PD [37,38,39]. Considering that anxiety is the pathogenic overlap of PD, this linkage highlights the intricate interplay between the GI disorder and the pathogenesis of PD [39]. Cys-HCl administration has been reported to impair dopamine synthesis in the GI tract, resulting in gastric ulceration leading to affective and neurodegenerative disorders [7]. The degeneration of dopamine-producing neurons in the SN and OB is a prominent neuropathogenic hallmark of PD [40,41]. As a consequence, reduced levels of dopamine in the brain have been directly linked to motor deficits, olfactory dysfunction, and memory loss in PD [42]. Interestingly, S. Szabo and C.H. Cho reported that Cys-HCl exerts neurotoxicological effects similar to those exerted by the dopaminergic neurotoxin MPTP [10]. The conversion of MPTP into the toxic metabolite 1-methyl-4-phenylpyridinium (MPP)^+^ by monoamine oxidase-B in astrocytes, followed by the accumulation of MPP^+^ in dopaminergic neurons due to its high affinity for dopaminergic transporters in the SN, in turn, depletes adenosine triphosphate (ATP) and increases oxidative stress, leading to neuronal degeneration [43]. Adequate experimental evidence supports the monoamine oxidase inhibitory effect of Cys-HCl [44]. PD has been linked to the inhibition of mitochondrial monoamine oxidase, which might be highly relevant in the context of the reduced levels of dopaminergic neurons in the brains of Cys-HCl-treated mice [44,45]. The Cys-HCl administration-induced reduction in antioxidants in experimental brains has become increasingly evident [18]. The anticancer effect of Cys-HCl has been reported to be associated with mitochondrial degeneration linked to the accumulation of iron, and this event activates peroxidase-positive autophagosomes, ultimately leading to cell death [46,47]. Considering these facts, it has been proposed that Cys-HCl induces iron-dependent, lipid peroxidation-driven ferroptosis, a mechanism that could be linked to the neurodegenerative processes observed in PD [48]. Moreover, Cys-HCl is known to be a potent depigmenting molecule that reduces melanin synthesis through the inhibition of tyrosinase activity in the skin [49]. Strikingly, dopaminergic neurons generate a neuromelanin-like pigment, structurally similar to melanin, in the substantia nigra, which is associated with dopamine synthesis and is essential for neuroprotection [50]. Notably, the interaction of MPTP with neuromelanin is known to be a specific cause of the degeneration of dopaminergic neurons in the substantia nigra of the brain [51]. Considering the depigmentation nature of Cys-HCl, it can also specifically target neuromelanin-producing dopaminergic neurons in the SN and OB of experimental animals. Therefore, the reduced number of TH-positive dopaminergic neurons observed in the brains of the Cys-HCl-administered mice partly indicates the neuropathogenic signatures related to PD.

In contrast, a few studies have shown that Cys-HCl exhibits therapeutic benefits against transgenic and toxic experimental models of PD. For instance, Alberto Siddu et al. reported that Cys-HCl treatment increased the TH levels and improved the motor performance in a transgenic Thy1-α-Syn model of PD, which was characterized by the overexpression of human α-synuclein [52]. However, this specific Thy1-α-Syn mouse model has been characterized by longer lifespans with no change in the number of TH-positive neurons, and no clear clinical signs of behavioral deficits, neurodegeneration, or motor neuron pathology [53,54,55]. According to Linjuan Sun et al., Cys-HCl ameliorated MPTP-induced neurodegeneration and oxidative stress in a mouse model of PD, suggesting its neuroprotective role [56]. Indeed, these findings are surprising, given that Cys-HCl itself has been demonstrated to mimic the neurotoxic effects of MPTP. The reason for the contradictory reports on the effects of Cys-HCl remains to be established [10]. Potential explanations for this discrepancy could include differences in the experimental design, dosage, duration of administration, deferential production of metabolites, and validation procedures. Therefore, further studies are needed to clarify these confounding factors and to better understand the underlying mechanisms by which Cys-HCl exerts its beneficial or neurotoxic effects.

The physiological level of dopamine in the brain is crucial for the regulation of movement, motivation, mood, and cognitive functions [57]. As TH is an enzyme crucial for the synthesis of catecholamines, including dopamine, TH-positive neurons in the SN and OB substantia are often studied in the context of motor functions and olfaction [58]. It has been well established that the loss of dopamine-producing TH-positive neurons in the brain leads to a range of motor and non-motor symptoms, including olfactory bulb (OB) deficits, which are neuropathogenic characteristics of PD [42]. Based on the impairment of motor, olfactory, and cognitive behavioral outcomes in correlation with reduced dopamine neurons addressed by immunohistochemistry, the current study suggests that the neurotoxicological effects of Cys-HCl recapitulate the key clinical symptoms and pathological features of PD. The possible adverse effects associated with Cys-HCl treatment practiced for various disorders may not be excluded. This study warrants careful consideration and monitoring in clinical settings to ensure safety and optimize the therapeutic outcomes related to Cys-HCl, and the results reinforce the importance of considering GI defects and neurological defects related to PD.

## 5. Conclusions

Cys-HCl is a well-established chemical agent for inducing gastric ulcers in experimental animal models. Moreover, its depigmentation properties in the skin are also well established, as it interferes with melanin production by antagonizing the activity of the TH pathway. Hence, it can also impact the neuromelanin in dopamine-producing neurons in the brain. Results obtained from the behavioral studies show that Cys-HCl treatment impairs the motor, olfactory, and cognitive functions. In addition, Cys-HCl administration has been found to significantly reduce TH-positive cells in the SN and OB in experimental animals. This study provides robust evidence that Cys-HCl-induced GI disorder in mice results in significant neurobiological impairments, particularly mirroring the dopaminergic deficits and motor dysfunction seen in PD. Eventually, Cys-HCl treatment could be considered a model of experimental PD. At present, the possible adverse effects of Cys-HCl in humans need to be thoroughly evaluated and monitored to ensure patient safety and optimize therapeutic outcomes.

## Data Availability

The original contributions presented in this study are included in the article. Further inquiries can be directed to the corresponding author.
